# Spray-Coated MoO_3_ Hole Transport Layer for Inverted Organic Photovoltaics

**DOI:** 10.3390/polym16070981

**Published:** 2024-04-03

**Authors:** Hou-Chin Cha, Chia-Feng Li, Tsui-Yun Chung, Wei-Yang Ma, Cheng-Si Tsao, Yu-Ching Huang

**Affiliations:** 1 Department of Physics, National Atomic Research Institute, Taoyuan 32546, Taiwan; hccha@nari.org.tw (H.-C.C.); tychung@nari.org.tw (T.-Y.C.); pony@nari.org.tw (W.-Y.M.); tsaochengsi@gmail.com (C.-S.T.); 2 Department of Materials Engineering, Ming Chi University of Technology, New Taipei City 24301, Taiwan; d10527005@ntu.edu.tw; 3 Department of Materials Science and Engineering, National Taiwan University, Taipei 10617, Taiwan; 4 Organic Electronics Research Center, Ming Chi University of Technology, New Taipei City 24301, Taiwan; 5 Department of Chemical and Materials Engineering, College of Engineering, Chang Gung University, Taoyuan 33302, Taiwan

**Keywords:** inverted organic photovoltaics, MoO_3_ solution process, large-area spray coating

## Abstract

This study focuses on the hole transport layer of molybdenum trioxide (MoO_3_) for inverted bulk heterojunction (BHJ) organic photovoltaics (OPVs), which were fabricated using a combination of a spray coating and low-temperature annealing process as an alternative to the thermal evaporation process. To achieve a good coating quality of the sprayed film, the solvent used for solution-processed MoO_3_ (S-MoO_3_) should be well prepared. Isopropanol (IPA) is added to the as-prepared S-MoO_3_ solution to control its concentration. MoO_3_ solutions at concentrations of 5 mg/mL and 1 mg/mL were used for the spray coating process. The power conversion efficiency (PCE) depends on the concentration of the MoO_3_ solution and the spray coating process parameters of the MoO_3_ film, such as flow flux, spray cycles, and film thickness. The results of devices fabricated from solution-processed MoO_3_ with various spray fluxes show a lower PCE than that based on thermally evaporated MoO_3_ (T-MoO_3_) due to a limiting FF, which gradually increases with decreasing spray cycles. The highest PCE of 2.8% can be achieved with a 1 mg/mL concentration of MoO_3_ solution at the sprayed flux of 0.2 mL/min sprayed for one cycle. Additionally, S-MoO_3_ demonstrates excellent stability. Even without any encapsulation, OPVs can retain 90% of their initial PCE after 1300 h in a nitrogen-filled glove box and under ambient air conditions. The stability of OPVs without any encapsulation still has 90% of its initial PCE after 1300 h in a nitrogen-filled glove box and under air conditions. The results represent an evaluation of the feasibility of solution-processed HTL, which could be employed for a large-area mass production method.

## 1. Introduction

Bulk heterojunction (BHJ) organic photovoltaics (OPVs) have attracted great attention due to their advantages of low manufacturing cost, mechanical flexibility, and solution processibility [1,2,3]. The BHJ structure is formed by a blend of an electron donor and acceptor materials [4]. Recently, the power conversion efficiency (PCE) of OPVs has exceeded 19% due to the rapid development of donor and acceptor materials [5,6]. Despite the promising performance of OPVs, their stability is still a limiting factor toward commercialization. In conventional-structure OPVs, poly(3,4-ethylenedioxythiophene):poly(styrenesulfonate) (PEDOT:PSS) and low-work-function metals, such as calcium (Ca) and aluminum (Al), are used as the hole electron transport layer (HTL) and top electrode, respectively. However, the acid nature of PEDOT:PSS and air-sensitive metal top electrodes have been associated with long-term instability. Therefore, inverted-structure OPVs have been broadly studied to improve the stability of OPVs. In inverted OPVs, metal oxides, such as zinc oxide (ZnO), titanium oxide (TiO_x_), and cesium carbonate (Cs_2_CO_3_), are used as an electron transport layer (ETL) on the indium tin oxide (ITO) substrate. In addition, transition metal oxides, such as MoO_3_ and vanadium(V) oxide (V_2_O_5_) [7], and high-work-function metals, such as gold (Au) and silver (Ag), are used as the HTL and metal top electrodes, respectively. Transition metal oxides used as alternatives to PEDOT:PSS could effectively increase the long-term stability of OPVs [8,9,10,11,12,13,14]. As usual, these metal oxides are deposited via a vacuum deposition process, such as sputtering, thermal or electron beam evaporation.

To lower the manufacturing cost of OPVs, solution processing methods have been developed recently. Solution-processed metal oxides as the HTL of OPVs have demonstrated equal performance and stability compared to HTL deposition via a vacuum process [15,16,17,18,19]. In recent years, solution-processed HTLs utilizing various alternative materials have been developed for highly efficient OPVs. Yang et al. demonstrated that the solution processing of MoO_x_ (S-MoO_x_) without post-annealing treatment is an effective approach to produce annealing-free, alcohol-processable MoO_x_ anode interlayers in PM6:Y6-based inverted OPVs, achieving a high PCE of 15.2% [20]. Additionally, Song et al. showcased an annealing-insensitive, alcohol-processed MoO_x_ HTL that universally enables high-performance conventional and inverted OPVs. By utilizing the S-MoO_x_ HTL annealed between room temperature and 110 °C with a PM6:Y6 active layer, both conventional and inverted OPVs exhibited excellent PCEs, above 15.74%. Moreover, the S-MoO_x_-based conventional and inverted OPVs with a PM6:L8-BO active layer exhibited outstanding PCEs of 18.21% and 17.12%, respectively [21]. Alongside that, Sung et al. demonstrated the use of a solution-processed S-MoO_3_ HTL in inverted PTB7:PC_71_BM polymer solar cells, obtaining high PCE and excellent stability. Under damp–heat conditions of 65 °C/65% relative humidity (R.H.), the T_80_ value measured was 1350 h, and the T_90_ value for 85 °C/air was 4400 h. The S-MoO_3_ HTL also displayed excellent photo-stability under continuous illumination with AM 1.5G light [22]. Moving beyond MoO_x_-based materials, Ioakeimidis et al. introduced a solution-processed antimony-doped tin oxide (ATO) hole-selective contact produced by a spray pyrolysis route [23]. This exhibited exceptional optoelectronic properties and functionality within non-fullerene acceptor PM6:Y6:PC_70_BM inverted OPVs. Device implementation revealed that a 130 nm thick ATO is an ideal solution-based HTL for inverted OPVs, providing a similar PCE and light stability performance to that achieved with the commonly used thermally evaporated MoO_3_ HTL [23]. Furthermore, Teng et al. presented a facile and efficient strategy for preparing HTLs by simply mixing VO_x_ nanoparticle emulsions with a PEDOT:PSS solution to optimize interfacial properties and device performance in non-fullerene OPV devices. Devices based on TPD-3F:IT-4F BHJ active layers achieved a boosted performance with a PCE of 10.2% and better stability compared to devices with a typical PEDOT:PSS HTL [24]. Despite the elevated performance and improved durability induced by solution-based HTLs, the HTLs in the OPVs fabricated in the above-mentioned studies were all constructed using spin coating, which is incompatible with large-area processing. A solution-processable method for large-area mass production is necessary for further development and scaling-up purposes.

Due to rapid progress in PCE and stability, numerous studies have focused on the mass production of OPVs [25,26]. Among these in-line compatible deposition methods, spray coating is a simple, rapid, and low-cost technique that can deposit films on arbitrary substrates [27]. This research mainly investigated the optimization of sprayed photoactive layer films by controlling the host solvent, spraying parameters, and post-treatment. Our previous study demonstrated that a high performance of 3.73% could be achieved. Regarding other interfacial buffer layers in OPVs, ETL and HTL are often solution-processed and compatible with the spray process [28]. For ETL, much literature showed the possibility of realizing efficient inverted OPVs by spray coating the ETL with zinc oxide or titanium oxide. The most common HTL employed with spray coating is PEDOT:PSS; other HTLs such as MoO_3_, VO_x_ and graphene oxide (GO) have been successfully deposited by the spray process. These HTLs have mainly been applied in the conventional structures OPVs, i.e., deposited on the ITO substrate; however, little study has been carried out on HTLs spray-coated on the photoactive layer (inverted structure). The reason for this phenomenon is that the coating of the charge transport layer using a solution process is carried out on the ITO in the conventional structure. The ITO can be pre-treated to achieve the expected hydrophilicity/hydrophobicity. However, when surface treatment is applied to the hydrophobic active layer film for favorable hydrophilicity, it is necessary to consider whether the treatment process will cause damage to it, which is also the focus of this study.

In this study, we present fully sprayed inverted OPVs, with all films, including ZnO, P3HT/PCBM, and MoO_3_, deposited by spray coating, except for the metal top electrode. We simply dispersed the MoO_3_ solution in isopropanol at various concentrations as the spray solution. The interfacial contact between the sprayed hydrophilic MoO_3_ and hydrophobic photoactive layer plays a critical role in the performance of OPVs. Therefore, we optimized the spray parameters to improve the interfacial contact, thus enhancing the PCE of inverted OPVs. The proposed method utilizes the sprayed HTL onto the active layer, and the S-MoO_3_ cells were compared to those with thermally evaporated MoO_3_. Atomic force microscopy (AFM) was conducted to clarify the interfacial contact between the HTL and the electrode. Furthermore, the stability of non-encapsulated OPVs in ambient air was investigated to ensure the lifetime of the solution-processed HTL OPV cells. Additionally, the solution-processed HTL method not only reduced fabrication time due to the non-vacuum process, but also can be implemented over a larger processing area, facilitating mass production to meet industry demands.

## 2. Experiments

### 2.1. Materials

Indium tin oxide (ITO)-coated glass substrate as the transparent electrode was purchased from Optical Filter Ltd. (Thame, UK) (EMI-ito 15, surface resistance of 15 Ω/square). The ITO glass was cleaned with acetone (Acros, Geel, Belgium, Mw: 58.08, 99%) and IPA (Acros, Mw: 60.1, 99%) sequentially in an ultrasonic tank. The sol-gel ZnO precursor was prepared from zinc acetate (Acros, Mw: 219.5, 99%) and ethanolamine (Sigma-Aldrich, St. Louis, MO, USA, Mw: 61.08, 99%). To prepare the ZnO precursor solution, we dissolved zinc acetate (1 g) and ethanolamine (0.28 g) in 10 mL of 2-methoxyethanol (Alfa Aesar, Ward Hill, MA, USA, Mw: 76.09, 99%). The formed pristine ZnO was then diluted with IPA at a volume ratio of 1:10. P3HT (Mw: 30–40 k, PDI: ~2.0), and PCBM (Mw: 910, 99.5%) was obtained from Rieke Metals (Lincoln, NE, USA). The P3HT and PCBM in 1:1 wt/wt ratio were dissolved in o-xylene (Alfa Aesar, Mw: 106.2, 99%) and stirred at 50 °C overnight for preparing the photoactive layer solution. For the preparation of the S-MoO_3_ solution, 0.1 g of molybdenum powder was dispersed in 10 mL of ethanol in an ultrasonication bath for 30 min. Subsequently, 0.35 mL of 30 wt% hydrogen peroxide was added to the solution to obtain hydrogen transition metal as the dried powder of S-MoO_3_ [22]. The dried powder was then dissolved in 10 mL of ethanol and stirred for 1 day. To ensure stable solution conditions and good coating quality of the sprayed film, IPA was added to the S-MoO_3_ solution. The S-MoO_3_ solution was then further diluted in IPA at the concentrations of 1 mg/mL and 5 mg/mL, and stirred overnight for the spray process.

### 2.2. Device Fabrication

**Figure 1:**
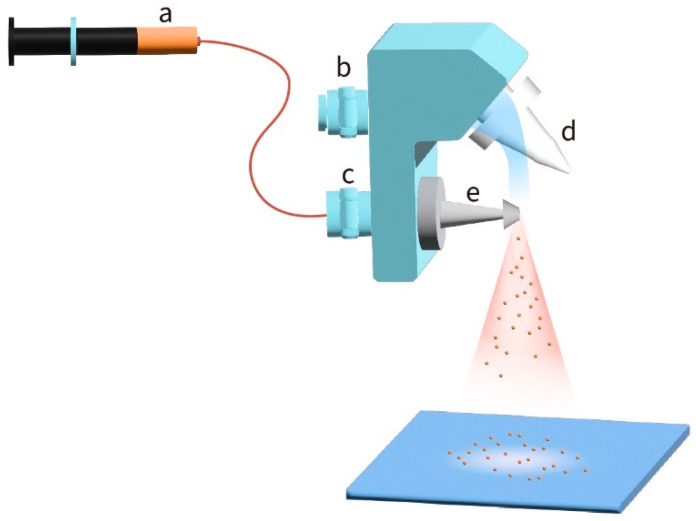
Schematic diagram of a spray coater. (a) The auto-solution injection controller regulates the flow rate of the injected solution; (b) the air inlet enables the flow of air into the air deflector; (c) the solution inlet allows the solution to be injected into the ultrasonic nozzle; (d) the flat jet air deflector adjusts the air stream pressure to control the impact of the atomized spray on the substrate; and (e) the ultrasonic nozzle atomizes the solution into droplets.

**Figure 2:**
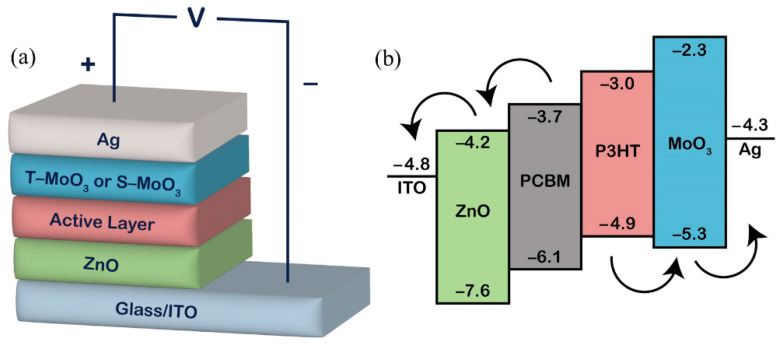
(**a**) A schematic representation of a device with an inverted structure; (**b**) the energy level diagram of the materials used in this inverted device.

The sol-gel ZnO precursor was sprayed onto the cleaned ITO glass substrate using an ExactaCoat system equipped with an ultrasonic atomizing nozzle (Sono-Tek Corporation, Milton, NY, USA). The ExactaCoat system is equipped with an AccuMist 120 kHz ultrasonic atomizing nozzle. Figure 1 depicts the schematic diagram of this system, which integrates the ultrasonic atomizing nozzle with a controlled jet of air from the flat jet air deflector. An auto-solution injection controller (Figure 1a) regulated the flow rate of the solution injected into the ultrasonic nozzle through a solution inlet (Figure 1c). The ultrasonic nozzle (Figure 1e) atomized the solution into droplets. An air flow is provided from the inlet, as illustrated in Figure 1b. Since the droplets randomly leave the ultrasonic nozzle, a guided air flow provided from the bottom of Figure 1d forced the droplets onto the substrate. The ExactaCoat system was outfitted with three stepper motors for controlling the ultrasonic nozzle at a desired position, and the total horizontal work area was about 30 cm × 30 cm, with a vertical distance of about 15 cm. The programming system allowed the users to set up the path in advance, enabling us to establish the correlation between the coating parameters and film thickness. The ZnO layer was calcined at 150 °C for 1 h. Then, the photoactive layer solution consisting of P3HT and PCBM was spray-coated on the ZnO layer. The sprayed photoactive layer was annealed at 130 °C for 10 min in air. Different MoO_3_ layers as HTL were deposited on the annealed photoactive layer by thermally evaporated and spraying processes. These devices were named T-MoO_3_ (thermally evaporated) and S-MoO_3_ (sprayed), respectively. Finally, a metal electrode of Ag (Admat, Norristown, PA, USA, Mw: 107.87, 99.995%) was thermally evaporated through a shadow mask on the MoO_3_ layer to prepare the devices with an area of 0.3 cm^2^ (1 × 0.3 cm^2^). Figure 2 shows the inverted OPV structure used in this study and the corresponding energy band gap diagram.

### 2.3. Device Characterization

The photovoltaic parameters were determined from the current density–voltage (J-V) curves measured under AM 1.5G illumination (100 mW/cm^2^) using a solar simulator (Mode#11000, Abet technologies, Milford, CT, USA) and a digital source meter (Keithley 2400) in air. More than 10 devices were fabricated and characterized in each batch. The morphology of the thin films with the inverted structure glass/ITO/ZnO/P3HT:PCBM/MoO_3_ were also measured by atomic force microscopy (Nanoscope III, Digital Instruments, Tonawanda, NY, USA). The thickness of the thin film was measured using a stylus profiler (AlphaStep D-100, KLA Tencor, Victor, NY, USA).

## 3. Results and Discussion

**Figure 3:**
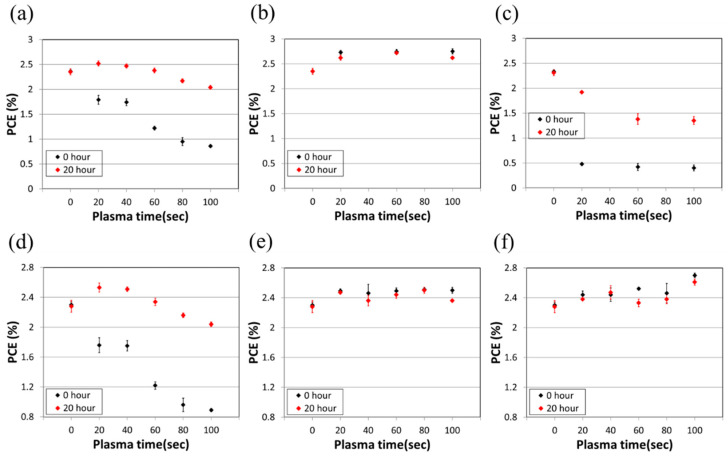
The effect of plasma treatment time on the PCE of devices with three types of plasma: (**a**) air, (**b**) N_2_, and (**c**) O_2_. The PCE (%) and plasma treatment times (s) for three types of power: (**d**) 18 W, (**e**) 12 W, and (**f**) 6 W, which were used in the plasma processes.

To enhance the efficient deposition of S-MoO_3_ onto the top of the active layer, three types of plasma treatments, including N_2_, O_2_, and air, were utilized before the spray coating process. The effects of the plasma treatment time on the PCE of the devices are illustrated in Figure 3. In the case of the air plasma treatment, all plasma times (20, 40, 60, 80, 100 s) exhibited lower PCE and instability, both at the initial value and after 20 h of storage in air, as depicted in Figure 3a. In the O_2_ plasma treatment, the PCE was also lower, showing poor stability at the initial value and after 20 h of storage in air, particularly with the plasma times of 20, 60, and 100 s, as illustrated in Figure 3c. Conversely, the use of N_2_ plasma treatment demonstrated a high PCE at the initial value and good stability after 20 h of storage in air, across the plasma times of 20, 60, and 100 s, as shown in Figure 3b. Moreover, the plasma power effect on the PCE of devices was also taken into consideration in this investigation, as shown in Figure 3d. The high-power (18 W) plasma process exhibited a low PCE at the initial value. However, the PCE showed improvement after 20 h of storage in air for all plasma times. In contrast, when medium power (12 W) and low power (6 W) were employed, both demonstrated favorable PCE and stability at the initial value and after 20 h of storage in air, as illustrated in Figure 3e,f. Specifically, the low power (6 W) at 100 s of plasma exposure achieved optimized PCE and stability, although requiring an extended plasma processing time. On the other hand, the medium power (12 W) at only 20 s of plasma exposure attained higher PCE and stability. Consequently, N_2_ plasma treatment was chosen for all subsequent device fabrication with medium power (12 W) with 20 s of plasma exposure.

**Figure 4:**
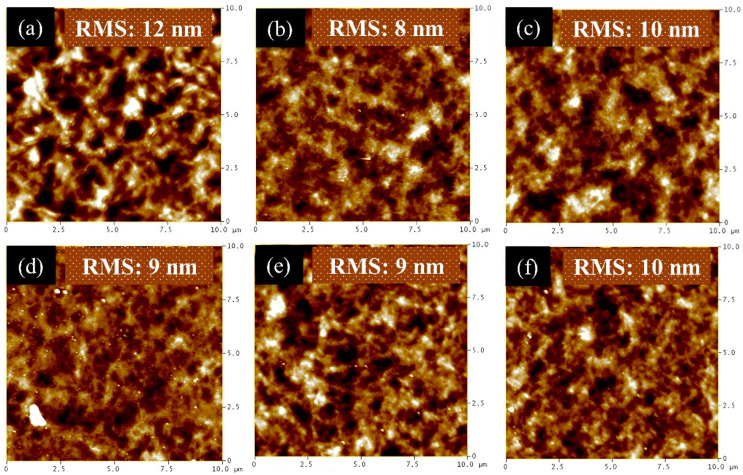
AFM images and RMS roughness of (**a**) the pristine spray-coated active layer and one cycle of the spray-coated HTLs with the following parameters: (**b**) 0.3 mL/min (5 mg/mL), (**c**) 0.1 mL/min (5 mg/mL), (**d**) 0.3 mL/min (1 mg/mL), (**e**) 0.2 mL/min (1 mg/mL), (**f**) 0.1 mL/min (1 mg/mL).

We thermally evaporated MoO_3_ HTL as reference devices. The tested devices were based on spray-coated MoO_3_ HTLs under various spraying conditions. There are two critical factors for the performance of inverted OPVs with the MoO_3_ HTL. One is the coverage of the MoO_3_ layer, and the other is the thickness of the MoO_3_ layer. A complete coverage of the MoO_3_ film over the photoactive layer could prevent direct contact between the metal electrode and the photoactive layer. Thus, the charge transport and FF are greatly improved for a high PCE. In addition to the film coverage, the thickness of the MoO_3_ layer also plays an important role in the performance of inverted OPVs due to its low conductivity. Therefore, we optimized the film coverage and thickness of sprayed MoO_3_ layers by controlling the concentration of the MoO_3_ solution and spray parameters, including the spray flux and cycles. The morphological images of the sprayed MoO_3_ on the photoactive layer measured by atomic force microscopy are shown in Figure 4. Figure 4a depicts the morphology of the sprayed photoactive layer. The root mean squared (RMS) roughness of the sprayed photoactive layer was 12 nm. At first, the MoO_3_ solution at a concentration of 5 mg/mL was spray-coated on the photoactive layer with a spray flux of 0.3 mL/min and a spray cycle of 1. After spray coating the MoO_3_ layer on the photoactive layer, the RMS roughness reduced from 12 nm to 8 nm, as shown in Figure 4b. The reduced RMS roughness indicates an improvement in the smooth morphology of the sprayed MoO_3_ layer deposited on the active layer. The smooth surface implies a good interfacial contact between the sprayed layers and the metal electrode. As the spray flux decreased from 0.3 mL/min to 0.1 mL/min, the RMS roughness increased slightly from 8 nm to 10 nm. Moreover, we decreased the concentration of the MoO_3_ solution from 5 mg/mL to 1 mg/mL. The AFM images of the MoO_3_ film sprayed with various spraying conditions are shown in Figure 4d–f. Figure 4d–f depicts similar morphologies compared to that of the film sprayed with the 5 mg/mL MoO_3_ solution. By tuning the sprayed condition (spray flux and cycles), the surface roughness varies within 2 nm. These results conclude that (1) the roughness of the sprayed MoO_3_ layers is lower than that of the sprayed P3HT:PCBM layer, and (2) the sprayed MoO_3_ layer demonstrates similar morphology and surface roughness prepared under these sprayed conditions. These sprayed conditions should affect the depositing thickness of the MoO_3_ layer. However, the film thickness was lower than 30 nm, so it could not be measured by the stylus profiler.

**Figure 5:**
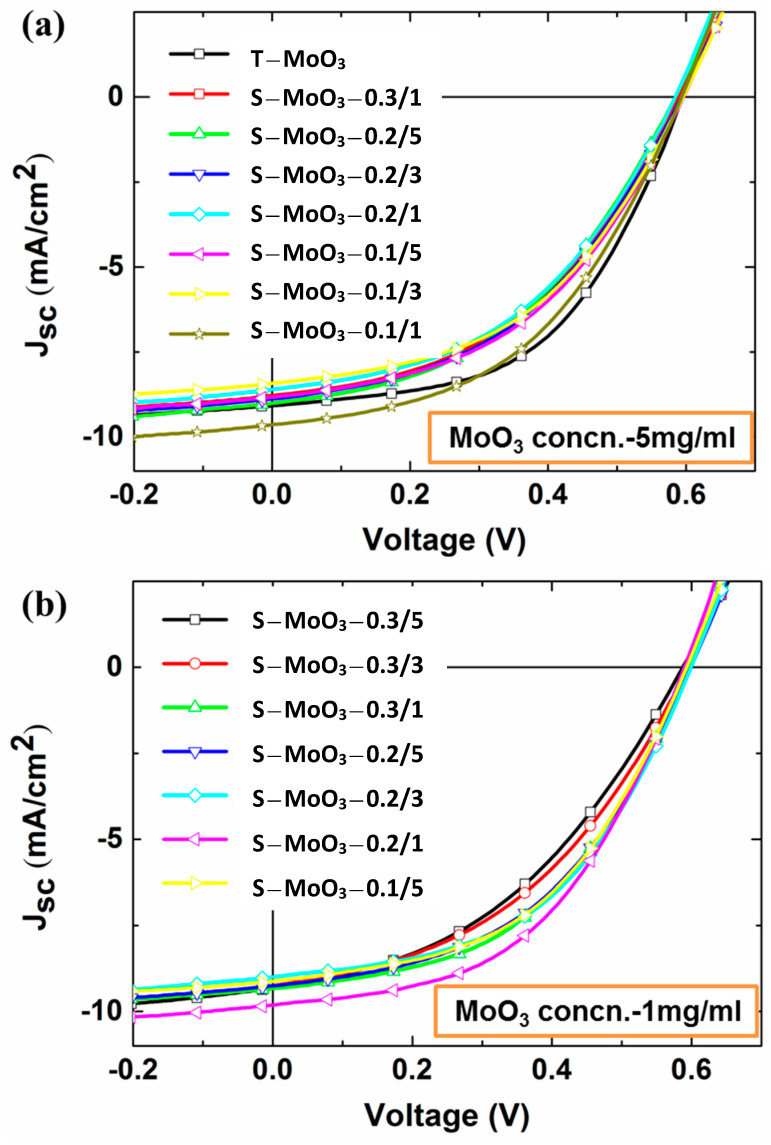
The J-V curves of the devices prepared with spray-coated MoO_3_ at the concentration of (**a**) 5 mg/mL and (**b**) 1 mg/mL.

Figure 5a shows the current–voltage (J-V) curves of the sprayed devices with thermally evaporated MoO_3_ layer (T-MoO_3_; reference device) and sprayed MoO_3_ film (S-MoO_3_) with a solution concentration of 5 mg/mL, respectively. The extrapolated photovoltaic characteristics of these devices prepared with different combinations of the spray flux and cycle number are listed in Table 1. In this study, the performance of inverted OPVs was averaged over 10 devices for each spray condition. The reference devices with the thermally evaporated MoO_3_ layer show an average PCE of 2.72%, short-circuit current density (J_sc_) of ~9.1 mA/cm^2^, open-circuit voltage (V_oc_) of ~0.59 V, and fill factor (FF) of ~50.3%. According to previous AFM results, we sprayed the 5 mg/mL MoO_3_ solution at the spray fluxes of 0.3 mL/min, 0.2 mL/min, and 0.1 mL/min combined with the spray cycles of 5, 3, and 1 to tune the sprayed MoO_3_ thickness. The resulting devices show a lower PCE than that of the reference device, mainly due to a limiting FF. It is important to note that the FF gradually increases with decreasing spray cycles. This result should be due to the decreasing film thickness. Moreover, the FF improvement usually implies that a reducing series resistance (*R_s_*) and a raising shunt resistance (*R_sh_*) were present in the devices. It is well known that the *R_s_* is strongly related to the interfacial contact resistance, and the *R_sh_* is mainly associated with recombination of the charge carrier. Therefore, this result indicates that the interfacial contact between the active layer and the sprayed MoO_3_ layer, or between the sprayed MoO_3_ layer and the metal electrode is improved by decreasing the spray cycles. In addition, the highest average PCE of 2.69% was achieved with the spray flux of 0.1 mL/min and one spray cycle, approaching that of the reference device. This result implies that the film thickness of the sprayed MoO_3_ layer is critical for the PCE and FF. Moreover, we changed the concentration of the MoO_3_ solution from 5 mg/mL to 1 mg/mL to study how the film thickness or quality can be tuned by the spray flux and spray cycles. The corresponding J-V curves and the electric characteristics are shown in Figure 5b and are listed in Table 1, respectively. At the spray flux of 0.3 mL/min, the devices with a thick MoO_3_ layer (5 cycles) showed a low PCE of 2.26%, mainly due to a limiting FF (41.05%). By reducing the spray cycles from 5 to 1, the PCE was improved to 2.59%, with an enhanced FF (47.70%). This is consistent with the above-mentioned results. Further, we optimized the combination of the spray flux and spray cycles. The highest PCE of 2.8% can be achieved with a spray flux of 0.2 mL/min and one spray cycle. The sprayed devices exhibited a similar FF compared to the reference devices. This result indicates that the conventional thermally evaporated MoO_3_ layer can be successfully replaced by the solution-processed MoO_3_ film. 

**Figure 6:**
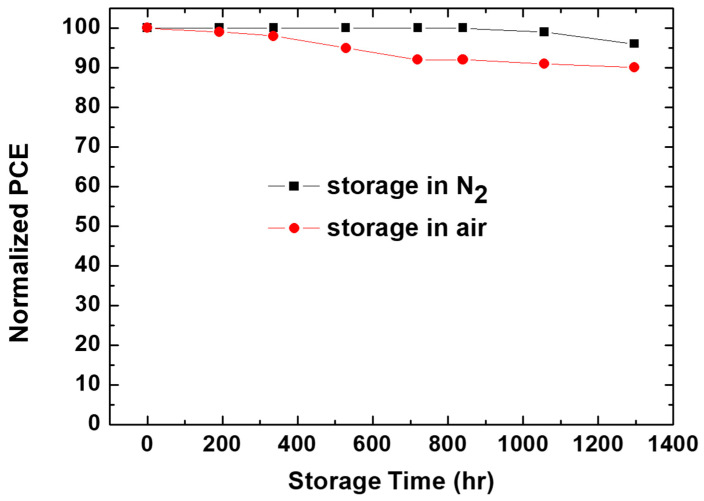
The stability curves of the non-encapsulated devices based on the sprayed MoO_3_ film in a nitrogen-filled glove box and under air conditions.

In Figure 6, the stability of non-encapsulated devices based on the sprayed MoO_3_ film, and a metal electrode of Ag is compared to devices based on the thermally evaporated MoO_3_ layer under shelf-life condition (ISOS-D-1 Shelf) [29]. The devices with the sprayed MoO_3_ showed remarkable stability, and this is in excellent agreement with the stability of the devices with a thermally evaporated MoO_3_ layer.

The devices retained 90% of the original PCE after 1300 h in air. Therefore, we can conclude that the sprayed MoO_3_ layer investigated here allows for devices with a comparable PCE and stability compared to those of the devices with thermally evaporated MoO_3_. 

## 4. Conclusions

This study mainly focused on the development of solution-processed MoO_3_ for inverted OPVs. We have successfully completed the fabrication of inverted OPVs, including the ETL, active layer, and HTL, using a spray coating process. We optimized the PCE of devices by adjusting the spray parameters, including the MoO_3_ concentration, spray flow rate, and spray cycles. By utilizing a spray coating process to fabricate the HTL of the OPV devices, comparable efficiency to devices fabricated using a thermal evaporation process for the HTL can be achieved. This indicates that the spray coating process proposed in this study for the HTL can successfully replace the thermal evaporation process for HTLs. Since the solution process can be applied to various large-area printing processes, it offers advantages for further scaling up or the module production of OPVs. In addition, OPVs fabricated using the spray coating process for the HTL exhibited a promising result of durability in ambient air, even without encapsulation, compared to the control groups placed in a nitrogenous environment. This is highly practical from the perspective of industrial mass production. Therefore, the development of a full spray coating process for the fabrication of OPVs can serve as a new option for developing high-durability, high-yield, and low-cost production techniques. Additionally, our results demonstrate the high commercialization potential of the sprayed-coated MoO_3_ layer for large-area OPVs.

## Figures and Tables

**Table 1:** Photovoltaic characterization of devices prepared with thermally evaporated and spray-coated MoO_3_ films. The device area is 0.3 (1 × 0.3) cm^2^, and the data are averaged over 10 devices.

Thermally Evaporated MoO_3_ Layer						
J_SC_ (mA/cm^2^)		Voc (V)		FF (%)	PCE (%)	PCE_max_(%)
9.10 ± 0.120		0.59 ± 0.003		50.30 ± 1.40	2.72 ± 0.071	2.81
Sprayed MoO_3_ Concentration = 5 mg/mL						
spray flux (mL/min)	spray cycle	J_SC_ (mA/cm^2^)	Voc (V)	FF (%)	PCE (%)	PCE_max_ (%)
0.3	1	8.54 ± 0.19	0.594 ± 0.006	45.20 ± 0.50	2.29 ± 0.077	2.42
0.2	5	9.20 ± 0.17	0.588 ± 0.007	42.25 ± 1.13	2.28 ± 0.035	2.33
	3	9.05 ± 0.26	0.588 ± 0.008	43.21 ± 0.98	2.30 ± 0.091	2.49
	1	8.48 ± 0.13	0.591 ± 0.004	45.18 ± 1.69	2.27 ± 0.092	2.42
0.1	5	8.94 ± 0.22	0.589 ± 0.006	43.91 ± 1.16	2.31 ± 0.093	2.50
	3	8.41 ± 0.25	0.591 ± 0.005	45.23 ± 1.23	2.24 ± 0.052	2.35
	1	9.80 ± 0.30	0.596 ± 0.004	46.13 ± 1.36	2.69 ± 0.059	2.79
Sprayed MoO_3_ Concentration = 1 mg/mL						
spray flux (mL/min)	spray cycle	J_SC_ (mA/cm^2^)	Voc (V)	FF (%)	PCE (%)	PCE_max_ (%)
0.3	5	9.35 ± 0.101	0.59 ± 0.002	41.05 ± 0.45	2.26 ± 0.020	2.28
	3	8.88 ± 0.424	0.59 ± 0.004	44.45 ± 1.56	2.34 ± 0.129	2.68
	1	9.11 ± 0.329	0.60 ± 0.005	47.70 ± 1.30	2.59 ± 0.063	2.67
0.2	5	9.54 ± 0.339	0.59 ± 0.007	45.40 ± 1.57	2.57 ± 0.153	2.97
	3	9.26 ± 0.339	0.60 ± 0.003	47.10 ± 1.35	2.61 ± 0.119	2.81
	1	9.64 ± 0.124	0.59 ± 0.008	49.60 ± 0.67	2.80 ± 0.063	2.85
0.1	5	9.22 ± 0.233	0.59 ± 0.004	47.01 ± 1.59	2.56 ± 0.081	2.97

## Data Availability

Data are contained within the article.
